# Advancing the Next Generation of Health Risk Assessment

**DOI:** 10.1289/ehp.1104870

**Published:** 2012-08-08

**Authors:** Ila Cote, Paul T. Anastas, Linda S. Birnbaum, Rebecca M. Clark, David J. Dix, Stephen W. Edwards, Peter W. Preuss

**Affiliations:** 1 U.S. Environmental Protection Agency, Washington, DC, USA; 2 U.S. Environmental Protection Agency, Research Triangle Park, North Carolina, USA; 3 Yale University, New Haven, Connecticut, USA; 4 National Institute of Environmental Health Sciences, National Institutes of Health, Department of Health and Human Services, Research Triangle Park, North Carolina, USA

**Keywords:** bioinformatics, molecular biology, NexGen, “omics,” risk assessment, systems biology

## Abstract

Background: Over the past 20 years, knowledge of the genome and its function has increased dramatically, but risk assessment methodologies using such knowledge have not advanced accordingly.

Objective: This commentary describes a collaborative effort among several federal and state agencies to advance the next generation of risk assessment. The objective of the NexGen program is to begin to incorporate recent progress in molecular and systems biology into risk assessment practice. The ultimate success of this program will be based on the incorporation of new practices that facilitate faster, cheaper, and/or more accurate assessments of public health risks.

Methods: We are developing prototype risk assessments that compare the results of traditional, data-rich risk assessments with insights gained from new types of molecular and systems biology data. In this manner, new approaches can be validated, traditional approaches improved, and the value of different types of new scientific information better understood.

Discussion and Conclusions: We anticipate that these new approaches will have a variety of applications, such as assessment of new and existing chemicals in commerce and the design of chemical products and processes that reduce or eliminate the use or generation of hazardous substances. Additionally, results of the effort are likely to spur further research and test methods development. Full implementation of new approaches is likely to take 10–20 years.

Risk assessment is a dominant public policy tool used to identify and evaluate scientific information to fulfill the missions of the U.S. Environmental Protection Agency (EPA) and other agencies by informing regulatory and technologic decisions, setting priorities for research, and supporting benefit–cost analyses [National Research Council (NRC) 2009]. The efficacy and timeliness of current risk assessment practices, however, are limited. Consequently, we are unable to evaluate the great number of new and existing chemicals, as well as emerging materials such as nanomaterials and biopolymers, entering the marketplace (NRC 2007; U.S. EPA 2009). Concomitantly, focus is increasing on the design and synthesis of less hazardous chemicals and processes, thus avoiding many environmental problems and fostering sustainability (Anastas and Eghbali 2010; Anastas et al. 2010; NRC 2011). We anticipate development and use of new higher throughput risk assessment methods to identify both safer and more toxic chemicals.

Several large, new health research efforts are developing approaches that use new technologies to modernize toxicity testing. Examples include Tox21 (Collins et al. 2008; Kavlock et al. 2009; U.S. EPA 2012a, 2012b), the National Institutes of Health’s (NIH) National Institute of Environmental Health Sciences (NIEHS 2011), the National Toxicology Program (a multiagency effort headquartered at the NIEHS) (Bucher et al. 2011), the U.S. EPA’s Chemical Safety for Sustainability research program (see Appendix 1), ToxCast™ (Dix et al. 2007; Judson et al. 2010a), and the Safety Evaluations Ultimately Replacing Animal Testing (SEURAT) research program (European Commission and European Cosmetics Association 2011). Of particular note is that the Tox21 program alone will generate new high throughput data on 10,000 chemicals, using > 100 assays, over the next few years.

Additionally, new European legislation, Registration, Evaluation, Authorisation and Restriction of Chemicals (REACH), aimed at ensuring chemical safety will generate substantial new “nonstandard” *in vitro* data (European Commission 2007; European Commission and European Cosmetics Association 2011). REACH legislation requires industry to provide information necessary for adequate evaluations of public health risks in response to concerns related to approximately 120,000 chemicals in European commerce, addressing a desire for increased assessment efficiencies and a reduced reliance on *in vivo* animal testing. Although REACH currently is generating mostly traditional data, the European Commission’s intent is to move expeditiously toward using new types of molecular and systems biology data, as illustrated by their 50-million euro, 5-year SEURAT research program (European Commission and European Cosmetics Association 2011). There is substantial overlap between the approximately 120,000 chemicals covered by REACH and those chemicals manufactured or used in the United States.

Although the ongoing efforts to develop new methods and data are significant, how risk assessments will incorporate this new information is not entirely clear. Consequently, the U.S. EPA has developed a program, Advancing the Next Generation of Risk Assessment (NexGen), which focuses on how to use this new information in hazard identification and dose–response assessment. Commensurate efforts required to advance exposure assessments are described elsewhere (Cohen Hubal et al. 2010; Egeghy et al. 2011).

## Objective and Methods

The objective of the NexGen program is to begin to incorporate recent progress in molecular and systems biology into risk assessment practice. A broad array of new data and methods is being considered, including genomics, epigenomics, transcriptomics, proteomics, and metabolomics. Initially, this effort will ensure that risk assessments include state-of-the-science information. The ultimate success of this program, however, will be based on the incorporation of new practices that facilitate faster, cheaper, and/or more accurate assessments of public health risks. We anticipate that these new approaches will have a variety of applications, such as the assessment of new and existing chemicals in commerce and the design of chemical products and processes that reduce or eliminate the use or generation of hazardous substances. The program, described briefly in this commentary, maps a course forward and engenders movement from strategy to practical application in risk assessment.

The NexGen program is a U.S. EPA–led, multiagency collaboration among the U.S. EPA, the NIEHS, the National Center for Advancing Translational Sciences, the Centers for Disease Control/Agency for Toxic Substances and Disease Registry, the Food and Drug Administration’s National Center for Toxicological Research, the Department of Defense, and the State of California’s Environmental Protection Agency. These agencies are pooling knowledge, data, and analyses to explore the use of new science in risk assessment and to provide advice to the U.S. EPA’s National Center for Environmental Assessment.

The broad set of questions we seek to address in the NexGen program is

How can these new data and methods substantively improve our understanding of risk?
Can scientifically sound assessments be made faster, cheaper, and/or more accurate using these new methods, and better address a variety of environmental management challenges (risk context)?
How can these new types of information best be incorporated into risk assessments and used to inform risk managers and the public?
What new policies and procedures are needed to produce consistent, reasonable, and robust assessments?

Specifically, NexGen aims to develop *a*) a NexGen framework informed by the NRC framework for risk-based decision making (NRC 2009); *b*) a bioinformatics system for knowledge mining, creation, and integration to serve risk assessment; and *c*) prototype assessments targeted to the risk context and iteratively refined through discussions with scientists, risk managers, and stakeholders. These three aims are discussed further below.

*Framework for risk-based decision making.* Developing and implementing new approaches to risk assessment will require engaging a broad spectrum of stakeholders. The NRC framework for risk-based decision making provides a structure for such stakeholder engagement. Key components of the framework are public stakeholder discussion in the problem formulation, scoping, and planning steps of risk assessment; increased transparency throughout the entire process; and tailoring risk assessments more closely to the risk context. The framework process provides opportunities for fostering transparent and open discussion among a broad array of stakeholders. This effort ensures access to a broad representation of stakeholders (not just experts in technical fields), fostering their desired level of understanding, meeting their specific information needs, and providing resources to less advantaged groups so that equal access to the process is guaranteed. In February 2011, the U.S. EPA and its NexGen partners held a public meeting to begin to engage stakeholders in the NexGen process (U.S. EPA 2011a). Additionally, an expert workshop, open to the public, was held by the Emerging Science for Environmental Health Decisions Committee of the National Academy of Sciences 14–15 June 2012.

**Figure 1:**
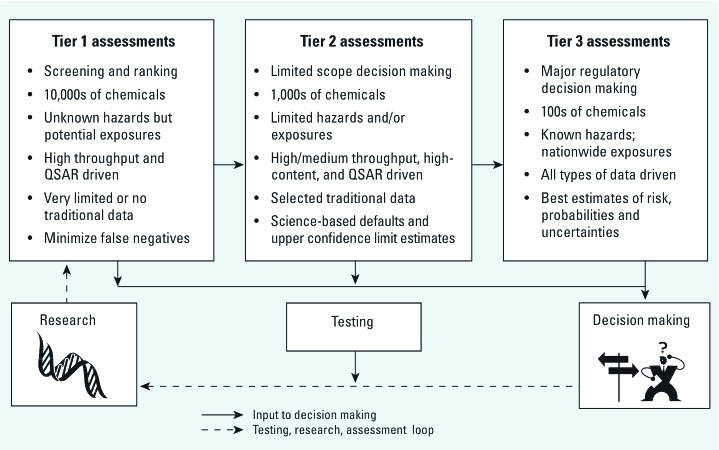
The proposed assessment paradigm is tailored to meet specific risk management needs for different types of environmental problems. From left to right, Tier 1 is designed to evaluate the tens of thousands of chemicals in commerce to which the American public is exposed, but for which we have little knowledge of hazard. Key to Tier 1 is rapid, inexpensive, high throughput biotechnology assays, coupled with quantitative structure–activity relationship (QSAR) analyses that allow screening and ranking of chemicals. Tier 2 is designed to evaluate hundreds of chemicals for which we have elevated concerns but limited traditional data, and it is intended to support limited-scope decision making. Key to Tier 2 is the use of both high and medium throughput bioassay data that provide some insight into tissue- and organism-level contributions to risk, as well as use of limited, conventional data. Last, Tier 3 targets the chemicals of national importance that are the focus of major regulatory decision making. Tier 3 uses all policy-relevant data, and is made more robust through the inclusion of molecular and systems biology knowledge.

An important task for NexGen is to match risk context to specific methodologies and to the level of scientific certainty required for decision making. To begin tailoring risk assessment approaches to the risk context, the NexGen program has constructed a three-tier scheme (Figure 1). Figure 1 shows distinct tiers with differing assessment approaches; in practice, these differing approaches lie on a continuum that could be modified for various situations. The cost of assessment in time, resources, and the number of animals used increases as one moves from Tier 1 to Tier 2 and then to Tier 3; scientific certainty also increases.

*Bioinformatics: knowledge mining, creation, and integration.* In today’s rapidly expanding world of information, productive use of new and existing information depends on the effective and efficient integration of dissimilar types of knowledge from a wide variety of sources. Information relevant to NexGen is found as unstructured information reported in the open literature, electronic “libraries” of molecular biology data such as those housed at the National Library of Medicine, and legally mandated test results reported to the U.S. EPA in rigidly structured formats. “Unstructured,” in this context, refers to how information is presented in the open literature text (e.g., not in a standardized format such as is done for test data submission) (Blake 2010). Consequently, we and others are developing informatics-based systems that support scientists as they face the daunting task of synthesizing diverse information from a wide array of resources. For example, diverse sources such as the U.S. EPA’s Aggregated Computational Toxicology Resource database, the NIH Comparative Toxicogenomic Database, and the National Library of Medicine Gene Expression Omnibus, in addition to textual descriptions of health end points found in hundreds of papers in the open literature, might contain, in combination, the necessary information to characterize hazard and exposure–response for a risk assessment. Informatics can help identify, summarize, and analyze large amounts of data from various sources for additional human consideration, as well as enable discovery and reduce the need to rely on known associations. The amount and breadth of data captured by informatics approaches will facilitate evaluation of both uncertainty (e.g., measurement error) and variability (e.g., among species, in humans), as recommended by the NRC (2009). Note, however, that informatics is a tool to assist scientists and not a replacement for human expertise and judgment.

*Prototype assessments.* A key feature of the NexGen program is the development of targeted prototype assessments to help engender movement from strategy to practical application. With these initial prototypes, we seek to demonstrate proof of concept, to characterize the value of information, and to determine decision rules for using new types of data and knowledge in risk assessment. We anticipate that the data-rich prototypes will *a*) help us understand how to use molecular and systems biology data to evaluate data-limited chemicals and *b*) provide insight into problematic issues generally unresolved by conventional data (e.g., response in the low-exposure range, characterization of a susceptible subpopulation). As part of this effort, we are exploring both qualitative and quantitative uses of the data and predictive methods and models (Chiu et al. 2010; Edwards and Preston 2008; Felter et al. 2011; Judson et al. 2011; Wetmore et al. 2012).

Although federal human health assessment guidelines explicitly encourage the use of mechanistic information, these guidelines largely reflect the knowledge and thinking of the 1980s and early 1990s. Currently, information concerning omic × environment interactions might be discussed qualitatively in assessments as supporting information, but, to date, such data have not been widely defined in regard to adversity (i.e., adverse or not adverse). Consequently, “omics” data have been used rarely in risk assessment and management decisions (Judson et al. 2010a; U.S. EPA 2011b).

Recent advances in scientific understanding of molecular and systems biology support the view that environmental chemicals can act through multiple toxicity pathways to induce adverse health outcomes (Edwards and Preston 2008; Guyton et al. 2009; Judson et al. 2010b, 2011; Miller et al. 2009). Moreover, the relationship between a dose and a particular outcome in an individual could take multiple forms depending on genetic background, target tissue, and other factors besides mechanisms of action. Interindividual variability and preexisting backgrounds of response are, in turn, key determinants of the population dose–response curve (NRC 2009). Moving from current risk assessment practices to risk assessment based on a modern view of disease will require a paradigm shift.

For the prototype human health assessments, we are evaluating several health end points/diseases at three levels of complexity, or tiers (Figure 1). Table 1 shows the prototype risk assessments currently under development. Prototypes will attempt to identify consistent molecular and cellular patterns reflective of causal relationships between chemical exposures and induction of human health end points and to evaluate exposure or dose relationships using these approaches. The intent is to use *in vivo* traditional data to explore further the predictive potential of both *in vivo* and *in vitro* “omic” data. Observed associations will be grouped into weight-of-evidence categories, describing the certainty with which an observed effect can be attributed to a particular chemical. In addition, each prototype will seek, to the extent feasible, to evaluate human variability, background health end point incidence, adaptation, and exposures to similar chemicals. Using such a construct, the effects of mixtures exposures and nonchemical stressors (e.g., socioeconomic factors, lifestyle) could be evaluated in later stages of the effort. Criteria for choosing the initial chemicals for prototype development were human exposures in which common, underlying mechanisms are generally understood, and both *in vitro* molecular biology data and *in vivo* traditional data are available for the chemical. We particularly emphasized the availability of *in vivo* human data, including observed responses at or near ambient concentrations and traditional upstream events. Initial work on methods used to inform the various tiers has been published (Judson et al. 2011; McHale et al. 2012; Thomas et al. 2011; Villeneuve et al. 2012). In partnership with the NIEHS, we are also adding diabetes/metabolic disease to the set of prototypes (not shown in Table 1) (Thayer et al. 2012). Over time, additional chemicals and health end point or disease combinations will be developed.

**Table 1:** Prototype risk assessments organized by issue

Issue	Lung injury and related respiratory disease	Endocrine disruption			Cancer
		Androgen	Thyroid		
Initial prototype chemicals	Ozone, chlorine	Phthalates	Bisphenol A, perchlorate	Benzene, benzo[a]pyrene, and other polycyclic aromatic hydrocarbons
Other related chemicals under consideration	Aldehydes, particulate matter, sulfur and nitrogen oxides, peroxyacetyl nitrate	Other biomonitored androgen hormone disruptors	Other biomonitored thyroid hormone disruptors	Other mutagenic and nonmutagenic carcinogens
Health end point/disease	Lung injury and related respiratory diseases	Testicular dysgenensis Reproductive dysfunction Fetal germ cell effects Malformations	Neurodevelopmental impairment	Cancer
Mechanisms of action	↑ Inflammation ↑ Airways reactivity	↓ Testosterone ↓ insl3	↓ Thyroid hormones	↑ Gene mutation ↑ Epigenetic changes ↑ Repair alterations
Sensitive subpopulations	Ozone-sensitive subpopulation, asthmatics, children	Fetuses, children	Fetuses, children	Fetuses, children
Exposure pathways	Air	Air, soil, water, food	Air, soil, water, food	Air, soil, water, food
Other stressors	Allergens, preexisting disease	Other anti-androgens, preexisting disease	Other disruptors, preexisting disease	Coexposures, preexisting disease
↑, increased; ↓, decreased.								

Underlying questions considered in these prototypes include the following:

How can molecular and systems biology provide insights into potential adverse effects, or a lack of effects, in humans—when combined with *in vivo* data or in the absence of *in vivo* data?
How can these data inform relative potency estimates or exposure/dose–response relationships predictive of *in vivo* human responses?
What is the role of dosimetry or physiologically based pharmacokinetic modeling in using *in vitro* data?
Can these data inform us about
  - Variability and susceptibility in the human population?
  - Mixtures interactions?
What are the strengths and weaknesses of these new approaches for assessing risks in the human population?
How can the probabilities of harm to public health be better characterized, including noncancer health effects, and how will uncertainty and variability be characterized?

Additionally, results of the prototype development efforts are likely to spur further research and test methods development.

## Discussion and Conclusions

The landscape of risk assessment is changing to such an extent that significant transformation of risk assessment is needed (NRC 2007, 2009, 2011; U.S. EPA 2009). These changes are driven largely by phenomenal advances in understanding the gene environment, the advent of several recent and important reports from the NRC, and volumes of new test data from U.S. and European efforts. These events are prompting us to look anew at risk assessment. With the efforts described in this commentary, we hope to begin to position the U.S. EPA thoughtfully for the future and to contribute to meaningful change within the larger risk assessment community. Hence, we are embarking on an exploration of new science and methods that can be incorporated into currently emerging and future risk assessments.

We describe here a program that is advancing the next generation of risk assessment by incorporating recent progress in molecular and systems biology into risk assessment. This is a U.S. EPA–led collaborative effort among several federal and state partners. The effort focuses on iterative development of the next generation of risk assessment prototypes, learning from these efforts, and then refining subsequent efforts based on this new knowledge. Resultant prototypes will guide the development of improved assessments within the U.S. EPA’s National Center for Environmental Assessment. We envision that these new methods will facilitate assessment of new and existing chemicals as well as the design of “greener” chemicals for a more sustainable future.

The NRC and others have stated that 10–20 years might be required before risk assessment can rely primarily on new advances in science. Crafting the changes needed for the next generation of risk assessment, however, should begin now.

## Appendix 1: NexGen is a component of the U.S. EPA’s Chemical Safety for Sustainability research program

Chemical safety is a major priority of U.S. EPA research. Moving toward a safer and more sustainable environment requires producing new and existing chemicals in safer ways. It means having the information and methods needed to make more informed, timelier decisions about chemicals, many of which have not been thoroughly evaluated for potential risks. U.S. EPA research on chemical safety is geared to meet this challenge.

Using innovative approaches, U.S. EPA scientists and their partners are embracing the principles of green chemistry to produce safer chemicals. They are also integrating a diversity of scientific disciplines to develop new prediction techniques, pioneering the use of innovative technologies for chemical toxicity testing, and designing tools to advance the management of chemical risks. Chemical safety for sustainability includes research in computational toxicology, nanotechnology, endocrine-disrupting chemicals, human health, and pesticides.

Chemical Safety for Sustainability research is focused on three main areas (U.S. EPA 2012a):

Providing scientific knowledge, tools, and models for integrated evaluation strategies
Improving assessment and informing management for chemical safety
Targeting high priority research needs for immediate and focused attention.

Results of this research will inform risk assessment as a tool for sustainability assessment and provide key input into sustainability decision making, thus enhancing “the ability to analyze present and future consequences of alternative decision options on the full range of social, environmental, and economic indicators” (NRC 2011).
